# Analysis of Preload of Three-Stator Ultrasonic Motor

**DOI:** 10.3390/mi13010005

**Published:** 2021-12-22

**Authors:** Zheng Li, Hui Zhao, Shuai Che, Xuetong Chen, Hexu Sun

**Affiliations:** School of Electrical Engineering, Hebei University of Science and Technology, Shijiazhuang 050018, China; zhaohuihbkd@163.com (H.Z.); cheshuaihbkd@163.com (S.C.); chenxuetong2020@163.com (X.C.)

**Keywords:** ultrasonic motor, preload, friction contact, modal analysis, output characteristics

## Abstract

The pre-pressure device of the ultrasonic motor plays a vital role in the design of the entire motor structure, the contact state of the stator and rotor of the motor, dynamic properties of the stator, friction and wear characteristics of the rotor; even the mechanical behaviors of the entire electric machinery have a profound impact. Appropriate pre-pressure is conducive to the smooth operation of the ultrasonic motor, so that the output performance remains excellent, reducing wear and effectively extend the service life of the motor. Therefore, the research on pre-stress is of great significance, as it can better optimize the structure of the three-stator ultrasonic motor and lay the foundation for the stable operation of the motor. First, this paper introduces the construction of the motor as a whole and the pre-pressure device briefly described the working mechanism of the motor, and then introduces the influence of the pre-pressure on the stator and rotor contact models, the position of the constant velocity point, and the modal frequency. Finally, the motor output under different pre-pressures is discussed. The performance experiment has determined the optimal pre-pressure interval, which provides help for its subsequent optimization.

## 1. Introduction

Over the past 20 years, the ultrasonic motor has developed rapidly, and it exists as a new pattern of mini-type special motor. Its working principle adopts the inverse piezoelectric effect of piezoelectric ceramics. It is compared with the traditional electromagnetic motor, and it is not interfered with by electromagnets. It provides a good guarantee for the normal operation of the maglev train motor under the nuclear magnetic resonance environment [1]. Ultrasonic motors have the advantages of low speed and large torque, small size and light weight, low noise operation, power-off and self-locking, and high and low temperature resistance [2,3,4]. They are widely used in the fields of aerospace, precision instrumentation, robot joint drive, medicine, etc., and have become a research hotspot for scholars. The ultrasonic motor is composed of a vibrating body and a moving body. The vibrating body, the stator, is composed of piezo-ceramics and a metal elastic body, and the moving body, the rotor, consists of an elastic body and a friction material. The stator is given an AC voltage excitation and made to work in the ultrasonic frequency band (greater than 20 kHz) to produce a slight vibration. This micro-mechanical vibration is then converted to rotation or linear motion through resonance amplification and friction coupling. It can be seen from its working principle that there are two kinds of energy conversion: electromechanical conversion and friction conversion [5]. According to the characteristics of wave propagation, ultrasonic motors have two typical motor types, namely traveling wave type and standing wave type [5,6]. The technology of ultrasonic motors is becoming more and more perfected, and both types have been used in commercial mass production.

As a new type of driver, it uses friction to drive the rotor to rotate after completing the conversion of electrical and mechanical energy. Preload refers to the axial squeezing force between the stator and rotor of the ultrasonic motor after assembly, its setting will have a greater impact on the contact state of the motor stator and rotor, the dynamic characteristics of the stator, the friction and wear of the rotor, and even the mechanical function of the entire motor output [6]. Proper pre-pressure helps the motor to run smoothly and ensure its good output performance. At the same time, it can also effectively alleviate the wear of the contact surface and reduce noise. The change of the pre-pressure will lead to the change of function of the motor. The control of the pre-pressure can change the maximum output torque and velocity of the motor during operation, and the overall efficiency of the motor will also change accordingly. Therefore, research on the pre-pressure of the ultrasonic motor has significance, and many domestic and foreign scholars have conducted in-depth research on their theoretical knowledge and experimental tests.

Zheng Jieji et al., through theoretical derivation, studied the effect of pre-pressure on the contact angle of the ultrasonic motor and the driving proportion in the contact area, and put forward the corresponding motor design optimization criteria through the experimental test results [7]. Guo Yong et al. used the vibration test and the theory of constant velocity points to establish a mechanical model of the motor, analyzed the optimal solution problem when the rotating ultrasonic motor is in motion, and the relevance between the optimal solution and the load [8]. Tomoaki Mashimo proposed a minimum preload generating device and verified the influence of the axial and radial preload mechanism on improving the performance of the motor through experiments [9]. Pirrotta et al. studied the influence of pre-pressure on frequency properties. By analyzing the sensitivity and comparing with the results of finite element modal analysis, they found that the first-order resonance frequency is not susceptible to the effect of pre-pressure [10]. Wang Guangqing et al. have shown through experiments that the operating temperature and mechanical performance of the ultrasonic motor are affected by the proper pre-pressure, and a reasonable pre-pressure value can make the motor performance the best [11]. Zhou Shengqiang used ANSYS’s three-dimensional point contact element to establish a spatial areas analytical model of the contact interface of a traveling wave ultrasonic motor in stable conditions [12]. The power output performance of the pre-pressure and the contact interface are obtained from the force–balance relationship [13,14].

This paper studies the pre-pressure device of the three-stator ultrasonic motor in our laboratory, studies its influence on motor characteristics, and finally obtains the optimal pre-pressure range through experiments.

## 2. Three-Stator Ultrasonic Motor Structure and Working Mechanism

### 2.1. Motor Structure

**Figure 1:**
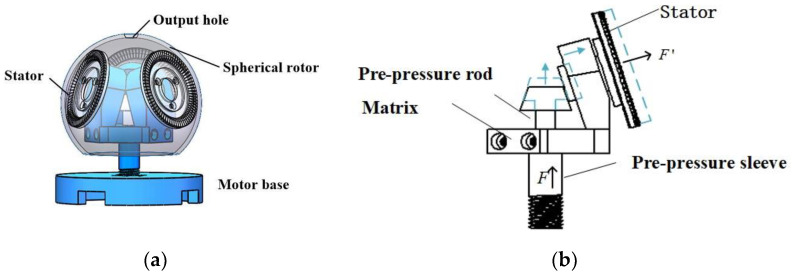
Schematic diagram of motor structure and pre-pressure device. (**a**) Motor structure. (**b**) Pre-pressure device.

Contrasted with the traditional motor with the stator outside, the prototype in this laboratory has higher integration, compact structure, smaller volume, and more convenient pre-pressure application. It uses three circular traveling-wave stators and a spherical rotor that is uniformly distributed in space and forms a 120° mutual plane. The main body is composed of a pre-pressure adjusting device, a bearing and a motor base, as shown in Figure 1.

The devising of the pre-pressure regulating device of the ultrasonic motor is very important, as it determines the overall performance parameters. The pre-pressure device of this machine is composed of a truncated cone type pre-pressure adjustment apparatus, a connecting rod, and a bushing, forming a T-shaped pre-pressure regulating device. In actual operation, by adjusting the tightness of the motor base and the pre-pressure adjusting device nut, the pre-pressure adjusting rod moves up and down to determine the magnitude of the pre-pressure applied.

**Figure 2:**
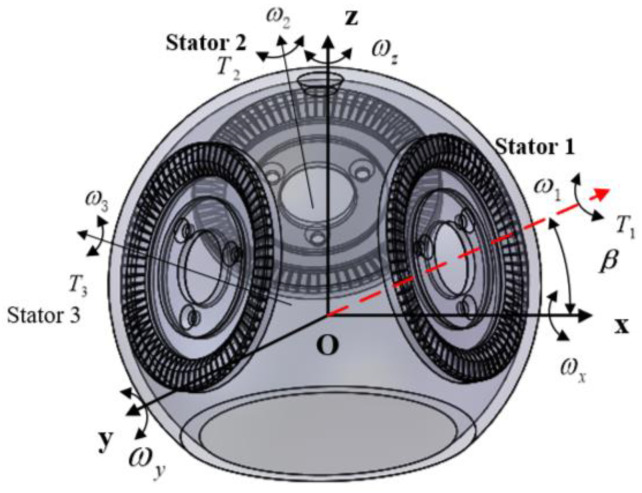
Angular velocity and torque distribution diagram of multi-degree-of-freedom spherical ultrasonic motor.

The surface of the traveling-wave stator has 72 laser-shaped stator teeth evenly distributed along the circumferential edge. The rotor contacts to drive the rotor to rotate. In order to make the stator and the rotor contact more fully under the action of pre-pressure, when designing the stator teeth, the outer edge of the teeth is chamfered by 0.5 mm × 45°. Furthermore, the inner surface of the globular rotor is coated with Teflon to strengthen the rub with the stator. The coplanar driving angular speed of the three stator directions is constant and can cooperate with each other to control the rotation of the spherical rotor in a certain direction. The combination of three traveling wave stators enables the motor to achieve three-degree-of-freedom movement. According to Figure 2, a spatial coordinate system is established, and the angle between the central axis of the three stators and the XOY plane is *β*. *ω*_1_, *ω*_2_, and *ω*_3_ are the angular velocity scalar, and *u*_1_, *u*_2_, and *u*_3_ are the corresponding direction vector. *u*_2_ and *u*_3_ are obtained by rotating u1 around the Z axis by 120° and 240°, respectively, so they are:

(1) $$u_{1}={\left[\begin{array}{ccc} \cos\beta & 0 & \sin\beta \end{array}\right]}^{T}$$

(2) $$u_{2}={\left[\begin{array}{ccc} -\frac{1}{2}\cos\beta & \frac{\sqrt{3}}{2}\cos\beta & \sin\beta \end{array}\right]}^{T}$$

(3) $$u_{3}={\left[\begin{array}{ccc} -\frac{1}{2}\cos\beta & -\frac{\sqrt{3}}{2}\cos\beta & \sin\beta \end{array}\right]}^{T}$$

So the angular velocity is:

(4) $$\omega_{s}=\left[\begin{array}{l} \omega_{x}\\ \omega_{y}\\ \omega_{z} \end{array}\right]=\left[\begin{array}{l} \omega_{1}\cos\beta -\frac{1}{2}\omega_{2}\cos\beta -\frac{1}{2}\omega_{3}\cos\beta \\ \frac{\sqrt{3}}{2}\omega_{2}\cos\beta -\frac{\sqrt{3}}{2}\omega_{3}\cos\beta \\ \omega_{1}\sin\beta +\omega_{2}\sin\beta +\omega_{3}\sin\beta \end{array}\right]$$

The output torque synthesized by the superposition of three stators is:

(5) $$T=\left[\begin{array}{l} T_{1}\cos\beta -\frac{1}{2}(T_{2}+T_{3})\cos\beta \\ \frac{\sqrt{3}}{2}(T_{2}-T_{3})\cos\beta \\ (T_{1}+T_{2}+T_{3})\sin\beta \end{array}\right]$$

### 2.2. Working Principle

**Figure 3:**
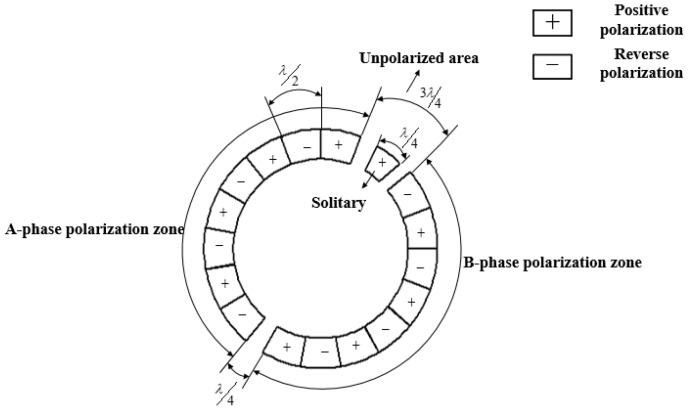
Schematic diagram of piezoelectric ceramic sheet.

Piezoelectric ceramics do not essentially exhibit piezoelectricity, and the piezoelectric effect can only be produced after they are polarized. According to the working principle of the ultrasonic motor, the second type of piezoelectric equation is selected:

(6) $$\left\{ \begin{array}{l} D=\varepsilon^{s}E_{1}+eS\\ T_{1}=-e^{t}E_{1}+c^{E_{1}}S \end{array}\right.$$

where, $T_{1}$ is the stress tensor, S is the strain, D is the potential shift tensor, $E_{1}$ is the electric field intensity, $c^{E_{1}}$ is the elastic coefficient under the constant electric field intensity, $\varepsilon^{S}$ is the constant dielectric coefficient, e is the piezoelectricity constant. The ultrasonic motor has the aid of converse piezoelectric effect of the piezoelectric ceramic to make the piezoelectric ceramic sheet work in the mode $d_{31}$. The two adjacent piezoelectric ceramic sheets have opposite polarization directions, which will respectively produce two kinds of opposite deformations of expansion and contraction. The stator elastic body does not produce this kind of expansion and contraction along with the piezoelectric ceramic sheet, but produces lateral bending deformation. When alternating excitation is given, this lateral bending deformation will form bending vibration in the stator elastic body. When only one excitation voltage is applied, this vibration will exist in the form of a standing wave. Piezoelectric ceramics are divided into A-phase polarization zone and B-phase polarization zone as shown in Figure 3. The length of each piece is λ/2 (*λ* is the wavelength), the radian is π/9, and there is an un-polarized area separated by 3λ/4 and λ/4 in the middle, where λ/4 as the isolated pole area. Although the isolated pole region is positively polarized, no voltage excitation is given to it, so it does not excite the stator; that is, it uses the positive piezoelectric effect. When the traveling wave is generated, the isolated pole region will also vibrate, which is used as a feedback pole to feed back the frequency tracking signal. If a closed-loop control is set in the isolated pole area, the deflection angle of the rotor can be accurately controlled.

The non-pitch-circle mode is adopted to ensure that the driving direction of each mass point of the stator tooth surface to the rotor is along the circumferential tangential direction, that is $B_{0n}$. This motor is designed in $B_{09}$ mode, and each traveling wave stator has nine waves. Given the two-phase A and B phases differ in space by π/2 mode shape function, the standing wave equation of the A-phase piezoelectric ceramic is:

(7) $$W_{A}(r,\theta ,t)=\xi_{A}\sin n\theta \cos\omega_{n}t$$

θ is the deflection angle, and *ω_n_* is angular frequency.

The standing wave equation of B-phase piezoelectric ceramics is:

(8) $$W_{B}(r,\theta ,t)=\xi_{B}\cos n\theta \cos(\omega_{n}t+\alpha )$$

where, $\xi_{A}$ and $\xi_{B}$ are the amplitudes of the A-phase and B-phase stators respectively, and α is the phase difference of the piezoelectric ceramics of the A and B phases in space. When two standing waves are superimposed and in α=π/2 and $\xi_{A}=\xi_{B}=\xi_{0}$, a forward traveling wave equation can be obtained:

(9) $$W(r,\theta ,t)=\xi_{0}\sin(n\theta -\omega_{n}t)$$

(10) $$\varsigma_{p}=\frac{H}{r}n\xi_{0}\sin(n\theta -\omega_{n}t)$$

*H* is the range between the mid-surface of the stator and the upper surface, *r* is the radius, and *n* is the number of modes.

So the tangential velocity of the particle is:

(11) $$V_{p}=\frac{d\varsigma_{p}}{dt}=-\frac{H}{r}n\xi_{0}\cos(n\theta -\omega_{n}t)$$

The size of the pre-pressure is adjusted by rotating the base of the electric machine. At a given value, the stator and the rotor contact to produce a frictional force. The relationship between the two is:

(12) $$f=\mu_{d}F$$

$\mu_{d}$ is the dynamic friction factor.

## 3. The Effect of Pre-Pressure on the Contact Range and Isokinetic Point of the Stator and Rotor

In order to form an effective friction pair between the stator and the rotor, pressure is applied to the normal of the contact surface of the two to make them contact. The magnitude of the pre-pressure has a great influence on the energy transmission of the motor, and has a far-reaching impact on its mechanical properties. Establishing a reasonable and effective contact friction model is an important means to study the performance of the motor by the pre-pressure, and it is of great significance to lay the foundation for the structural optimization of the ultrasonic motor [15,16].

**Figure 4:**
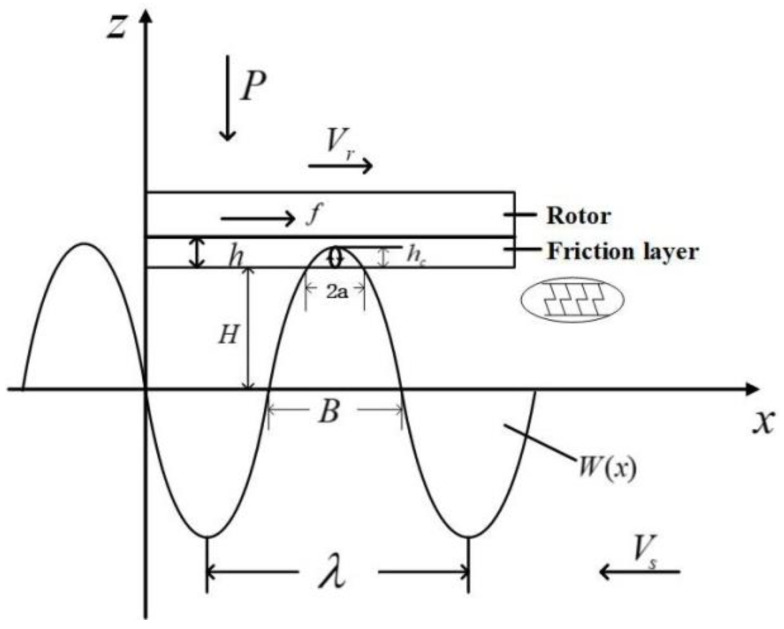
Contact model diagram of stator and rotor.

As shown in Figure 4, the particles on the stator surface move in the positive direction of the x-axis along an elliptical trajectory in the range of contact 2a. The particle will produce a displacement in the positive direction of the z-axis under the excitation of the traveling wave. As a result, the friction material on the inner layer of the rotor is squeezed to deform it, that is, a squeezing force *F_N_* along the z-axis is applied to the friction material. At the same time, the particle moves relative to the rotor. Within the contact range, there is a frictional driving force *F* between the two in the positive x-axis direction, the magnitude of which is *f*, which drives the rotor to move in the positive x-axis direction. The thickness of the friction layer is set to h, which can be ideally equivalent to a linear spring. From Hooke’s law, the normal force of the stator on the rotor per unit length can be obtained:

(13) $$\left\{ \begin{array}{l} f(x,t)=k\Delta x\\ k=\frac{EI}{L} \end{array}\right.$$

Among them, *k* is the equivalent spring stiffness, *E* is the elastic modulus of the spring material, and *I* is the geometric parameter formed by the spring size, *L* is the total length of the spring. The combination of the two can be obtained:

(14) $$f(x,t)=\frac{EB}{h}\Delta x$$

*B* is the radial width, and Δx is the amount of elastic deformation.

The stator dynamics equation of this motor is:

(15) $$M_{S}\ddot{x}+C_{s}\dot{x}+K_{s}x=\Theta V-F_{n}$$

*M_s_*, *C_s_* and *K_s_* are the modal quality, damping, and modal rigidity of the stator, separately, *x* is the displacement of the neutral plane, Θ is the electromechanical coupling coefficient, *V* is the excitation voltage, and *F_n_* is the pressure of the contact interface that is positively correlated with the pre-pressure. According to this formula, when the given pre-pressure increases, the contact range increases correspondingly; that is, the longitudinal restraint effect of the contact interface on the stator increases, so the amplitude of the stator will decrease. The fitting numerical relationship between the amplitude of the particles and the pre-pressure [11] can be expressed as:

(16) $$\xi =c\times F^{-m}$$

ξ is stator amplitude, *c* and *m* are constants.

In the positive direction of the z-axis, the rotor will receive downward pre-pressure and the upward squeezing force of the traveling wave generated by the stator on the rotor. The balance of the two forces can be obtained:

(17) $$F=n\int_{-a}^{a}\sigma_{z}(\theta )Bd\theta =\frac{nEB}{h}\cdot \int_{-a}^{a}\left(A\cos\theta -\zeta_{0}\right)d\theta$$

*n* is the number of traveling waves, *B* is the radial width of the contact area, $\zeta_{0}$ is the longitudinal displacement of the rotor base, and $\left(A\cos\theta -\zeta_{0}\right)$ is the deformation amount of the friction layer in the contact area.

**Figure 5:**
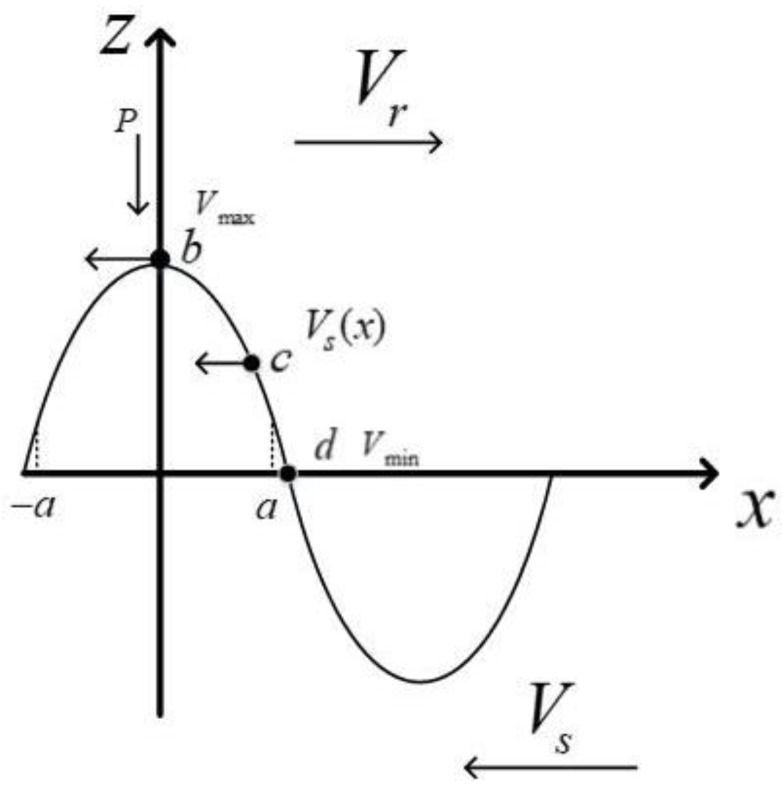
Schematic diagram of the velocity of the mass point on the stator superficies at different contact positions.

When the rotor runs at a constant speed, the horizontal speed of each mass point in the stator surface and its contact area is different. As shown in Figure 5, it runs at the maximum speed *V_max_* at point b, and runs at the minimum horizontal speed *V_min_* at the contact end *x* = *a*. The horizontal speed of the particles is also different at different contact positions [17,18,19,20,21]. When running with load, the rotor will run at a fixed speed, so there is a speed difference between the stator and the rotor outside the two constant velocity points. In the area [b, c], the stator surface particle speed is always greater than the rotor speed. In actual operation, this area can produce driving torque; in the [c, d] area, the rotor speed is always greater than the stator surface particle speed. In fact, the stator will obstruct the movement of the rotor will generate friction torque. The available symbolic functions are expressed as:

(18) $$\mathrm{sgn}\left(V_{s}-V_{r}\right)=\left\{ \begin{array}{l} 1,V_{s}>V_{r}\\ -1,V_{s}<V_{r} \end{array}\right.$$

Under the action of the pre-pressure, the stator and the rotor contact and generate an interaction force. The shape and force of the contact area do not change with time during the contact process.

The expression of the traveling wave representation of the stator neutrosphere is:

(19) W(x)=ξcos(kx)

When the rotor is running at a certain speed, the position function of the constant velocity point is:

(20) $$V_{s}(x)=k^{2}HC_{T}\xi \cos(kx)=V_{r}$$

*C_T_* is the propagation speed of the bending wave in the beam.

The positive pressure distribution on the stator surface is:

$$p_{0}(r,\theta ,t)=K_{m}h_{c}$$

(21) $$p_{0}(x)={\left(\frac{PE}{\pi Ra^{2}}\right)}^{\frac{1}{2}}{\left(a^{2}-x^{2}\right)}^{\frac{1}{2}}$$

Among them, *K_m_* is the bending stiffness of the rotor, and *a* is half the length of the contact area.

The force generated by friction contact is:

(22) $$f=\mu_{d}\iint_{\Omega }r\mathrm{sgn}p_{0}drd\theta$$

The driving torque is:

(23) $$M_{T}=fR=\mu_{d}\iint_{\Omega }R^{2}\mathrm{sgn}p_{0}drd\theta$$

Thus the effective output power of the ultrasonic motor is:

(24) $$P=M_{T}\omega_{r}$$

(25) $$N=\frac{30}{\pi }$$

(26) $$P=\frac{\pi }{30}M_{T}N$$

The sliding friction loss is:

(27) $$P_{slip}=\mu_{d}\iint_{\Omega }p_{0}\mathrm{sgn}|V_{s}-V_{r}|drd\theta$$

The motor efficiency is:

(28) $$\eta =\frac{P}{P_{slip}+P}\times 100\%$$

In addition, the actual operation of the motor will produce some radial loss. Due to the small size of this loss, it has little impact on the overall output performance of the motor. In order to appropriately reduce this loss, it can be within a certain small range. Adjusting the pre-pressure can also reduce the radial displacement by increasing the friction factor and Poisson’s ratio to reduce the radial loss. The radial slip is also related to the dip angle of the three stators. If the inclination angle is small, the radial slip value is large. When the inclination angle is 75°, the circumferential driving ability is better, which can reduce the diameter well. Due to the influence of the slip, considering the structure of the motor and the size of the inner diameter of the rotor, the optimal tilt angle is finally determined to be 72.404° [18].

Increasing the pre-pressure within a certain range will expand the contact area, increase the deformation of the friction material, and change the efficiency and energy loss of the motor. When the pre-pressure is small, the contact area is mainly concentrated at the peak of the stator traveling wave. This contact area is the driving area. In case of large preload and the contact area outstripping the position of the constant velocity point, the area is an obstructive area, that is, it obstructs the rotation of the rotor. According to Equation (23), it can be seen that under a given friction material, when $\mu_{d}$ remains unchanged, the size of the driving friction force is proportional to the pre-pressure. Different friction materials have different parameters such as stiffness, modulus, and friction factor, which will lead to differences in the contact interface during contact and at the same time affect the kinetic energy transmission of the contact interface. The modulus of elasticity is an important parameter for studying the frictional contact of the ultrasonic motor, which will affect the output torque, speed, motor efficiency and other functions. When the friction material is soft, that is, when its elastic modulus is small, the deformation of the friction layer is larger, and the contact range is larger. At this time, the contact range is mainly in the driving area, which has a more obvious effect on the rotor, so the speed will be increased accordingly. Under the same stress, with the enlargement of elastic modulus, its elastic deformation will shrink [22,23,24,25,26]. The elastic modulus of the composite material is:

(29) $$E_{a}=1-V_{d}E_{n}+n\left[1-\frac{\tan\beta }{\beta }\right]V_{d}E_{d}$$

In the formula, *V_d_* is the weight fraction of the reinforcing material, *E_n_* is the elastic modulus of the matrix material, *n* is the correlation coefficient between the matrix and the filler arrangement, β is the correction coefficient, and *E_d_* is the elastic modulus of the reinforcing material. Among them, according to this formula, it can be seen that as the weight fraction of the reinforcing material increases, the elastic modulus of the composite material will also increase, but this must be increased within a certain reasonable range, otherwise the crystal structure properties of the material itself will be destroyed, resulting in the material itself losing elasticity and the performance of the elastic modulus of the composite material deteriorating.

**Figure 6:**
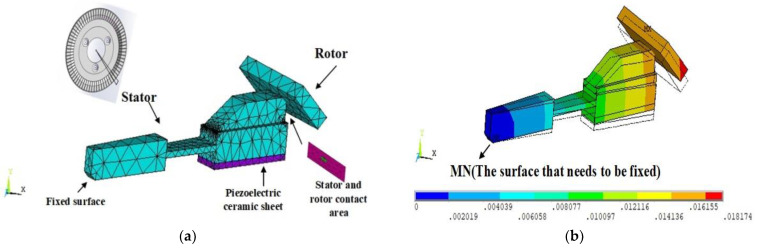
Schematic diagram of contact deformation of stator and rotor. (**a**) Stator and rotor contact modeling. (**b**) Stator and rotor contact simulation.

As shown in Figure 6, with the help of ANSYS software, we selected a tooth for modeling, fixed the surface at MN, and applied a force of 150 N on the bottom surface. The green area in (a) was the contact surface, and the contact friction coefficient was set to 0.2. In the simulation results, the rotor was warped due to the radial bending of the outer ring under the action of the pre-pressure, which caused the contact area to be significantly reduced, and the force was too concentrated, which will accelerate the contact surface wear, shortening the service life of the motor. For a mechanism based on ultrasonic motor, the axial pre-compression can ensure that the ultrasonic motor has a larger output torque.

In addition, the contact height *h_c_* should not exceed the distance between the axis of the traveling wave and the neutral plane, which will cause a sudden change in the curvature radius of the contact surface, and the forces in the tangential direction of the rotor will cancel each other out, thereby reducing the driving force obtained. The stator particles follow the law of conservation of momentum in the vertical direction, and the sum of the excitation force of the piezoelectric ceramic received by the particles and the driving force of the friction material is the momentum of the stator particles. According to the equivalent spring stiffness theory [27], it can be deduced that in the contact range a, the equivalent elastic modulus of the friction material is:

(30) $$E=\frac{Fh}{s}$$

*s* is the contact area.

## 4. The Influence of Preload on Modal Frequency

Pre-pressure affects both modal aliasing and modal frequency. Appropriate increase of pre-pressure can eliminate the phenomenon of modal aliasing [28,29].

(31) $$F=k_{d}w\left(x,t\right)$$

In the formula, *k_d_* is the equivalent elastic friction coefficient, and w(x,t) is the displacement of the stator’s transverse bending vibration. From this formula, it can be seen that the pre-pressure is proportional to the equivalent elastic friction coefficient, and its effect on the equivalent elastic friction coefficient is equivalent to the effect on the modal frequency. As the pre-pressure increases, the modal frequency of the stator will also be rise accordingly.

The simulation results can be used to verify and explain. This article uses ANSYS WORKBENCH software to realize the simulation of the stator model. The settings of the stator structure size and material coefficients of the motor during simulation are shown in Table 1 and Table 2.

**Figure 7:**
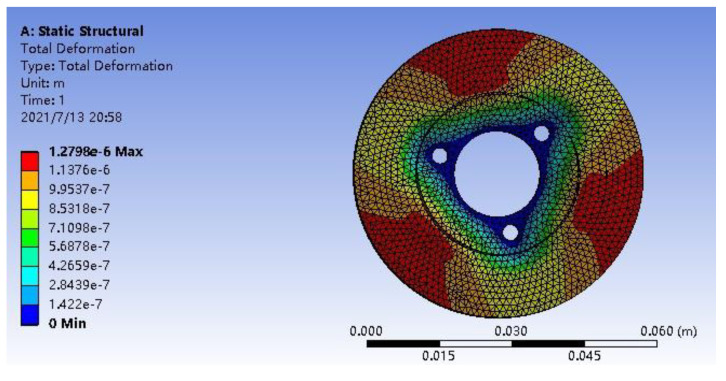
Equivalent total strain diagram of stator.

Firstly, through the static simulation analysis shown in Figure 7, it can be seen that the overall strain of the stator changes along the radial direction, the strain gradually increases, and reaches the maximum at the outer ring of the stator, and the stiffness and strength of the stator can meet the requirements.

(32) F=ps

*p* is the pressure, and *s* is the contact area.

**Figure 8:**
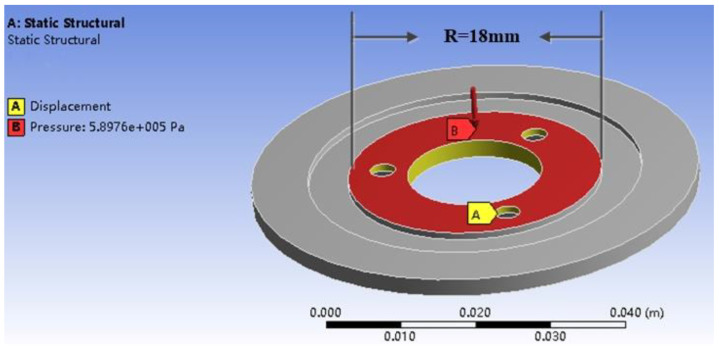
Schematic diagram of pre-pressure application.

As shown in the Figure 8, the yellow area is to fix the four screw holes on the stator in the three directions of x, y, and z, and the pressure values under different pre-pressures are applied in the red range. According to the size of the stator, we must calculate the area of the applied load, and then calculate the corresponding pressure value under the corresponding pre-pressure by Equation (32). The pressure value shown in the figure corresponds to 150 N.

**Figure 9:**
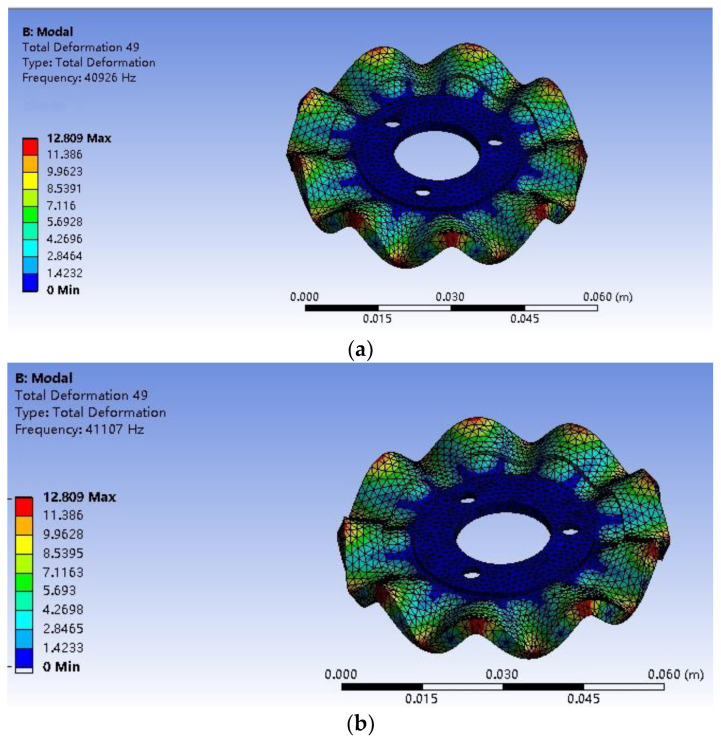
Stator modal frequency. (**a**) B_09_ mode frequency with a preload of 0 N. (**b**) B_09_ mode frequency with a preload of 50 N.

**Figure 10:**
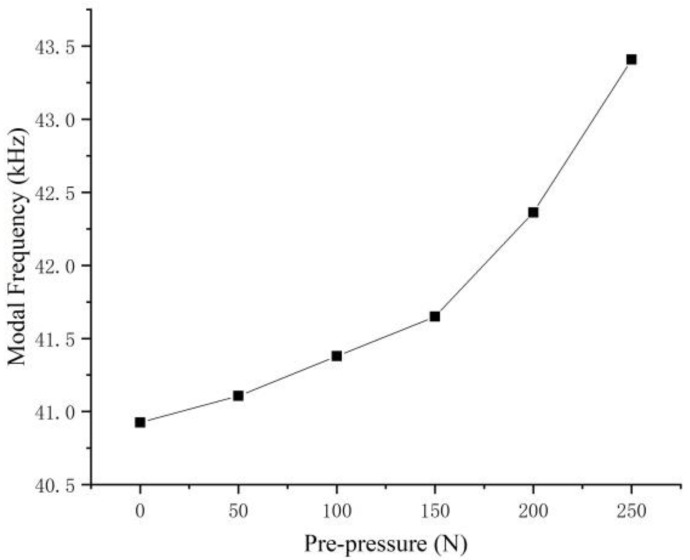
The relationship between preload and stator modal frequency.

Furthermore, the influence of a different preload on the modal frequency of the stator is studied by modal analysis. Specifically, the modal frequency is gradually increased from 0 N by 50 N to 250 N. Figure 9 shows the modal frequencies at 0 and 50 N. The result is shown in the Figure 10. It can be seen that during the increase of the pre-pressure, the overall stator modal frequency shows an upward trend, with a slow increase between 0–150 N and a rapid increase between 150–250 N.

**Figure 11:**
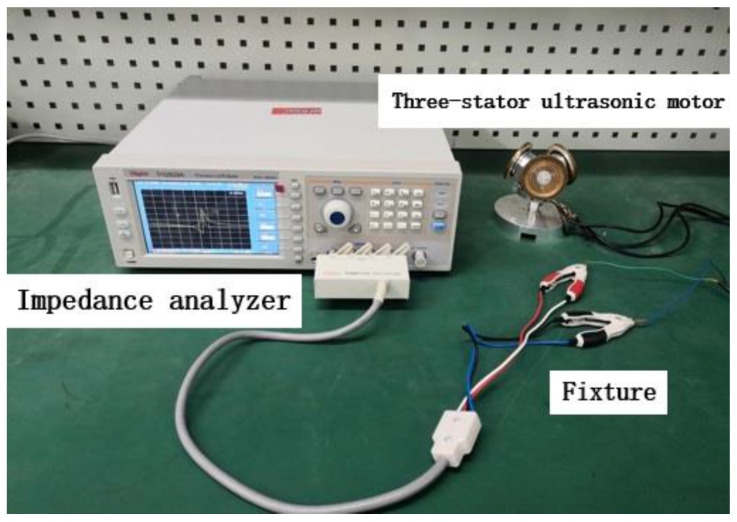
Schematic diagram of impedance analysis experimental device.

**Figure 12:**
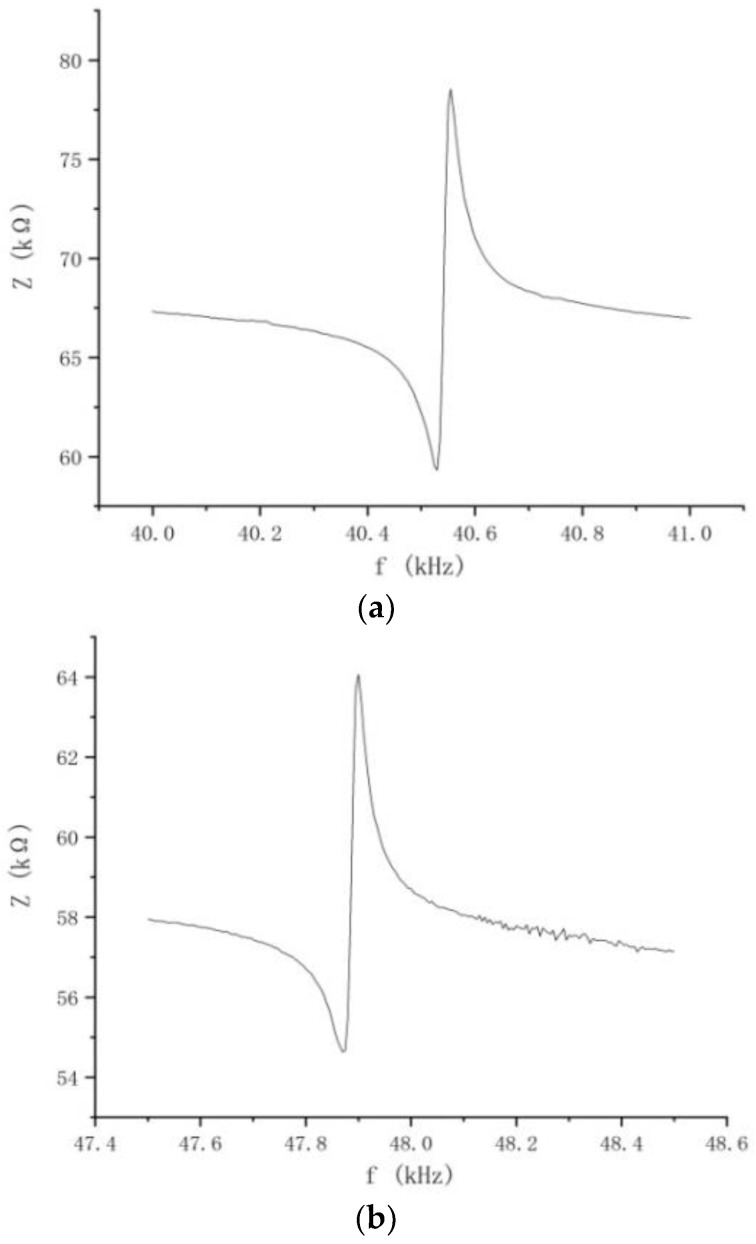
The effect of pre-pressure on frequency. (**a**) Impedance diagram when the pre-pressure is 0 N. (**b**) Impedance diagram when the preload is 500 N.

Then, we used the impedance analyzer in the experimental device shown in Figure 11 to scan the spectrum of one of the stators, and two clamps clamp the ground terminal and the voltage terminal respectively. The data are shown in Figure 12.

Comparing the two data graphs, as the pre-pressure of the motor increases, the frequency will increase, but the impedance value will be correspondingly smaller. The stator in the ultrasonic motor has a capacitive reactance characteristic, and the resistance value will become smaller as the frequency increases. It is because the increase of the pre-pressure will strengthen the restraint of the stator and rotor contact surface to the stator, resulting in the reduction of the stator’s amplitude [28]. However, the adjustment of the pre-pressure cannot be too large or too small. Too large will cause the axial force on the contact area to be too concentrated and wear [29]. Friction materials damage the rotor, and shorten the service life of the motor; a too small pre-pressure cannot reach the contact range, the stator cannot make frictional contact with the rotor, and the motor cannot run.

**Figure 13:**
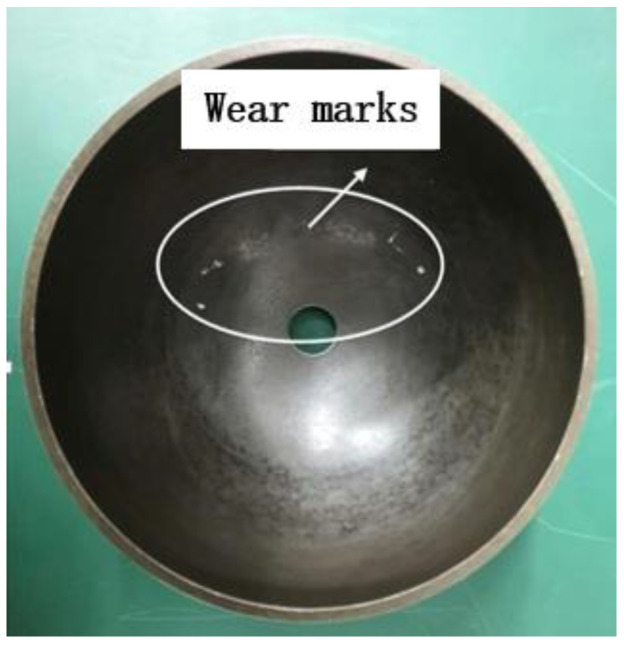
Schematic diagram of rotor wear.

Figure 13 shows the wear of the rotor friction material layer when the pre-pressure is adjusted incorrectly. When the wear is severe, the friction material layer will fall off, causing the rotor layer to be exposed and failing to protect the rotor. It can be observed from the data that the pre-pressure of 0 N is slightly different from the frequency result value obtained by the simulation and scanning through the impedance analyzer. This is because the pre-pressure device and the motor base are closely connected, and the base made of steel will increase the frequency accordingly. The error range is reasonable.

## 5. Experimental Test of Pre-Pressure on Motor Output Performance

**Figure 14:**
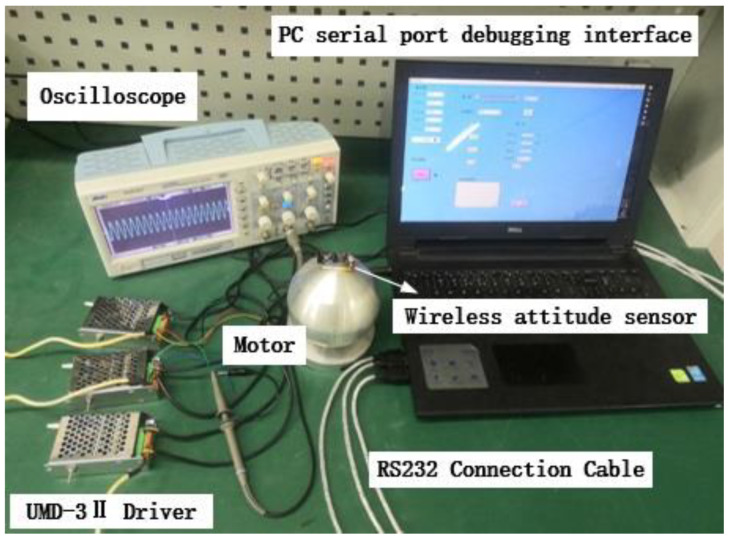
Output performance experimental test platform.

The experimental device shown in Figure 14 is used to test the effect of the pre-pressure on the speed at 150 N–250 N. Applying an excitation voltage of 400 V to the motor, the two-phase potential difference α ($\alpha =\frac{\pi }{2}$) and record the deflection speed of the motor through the wireless attitude sensor.

**Figure 15:**
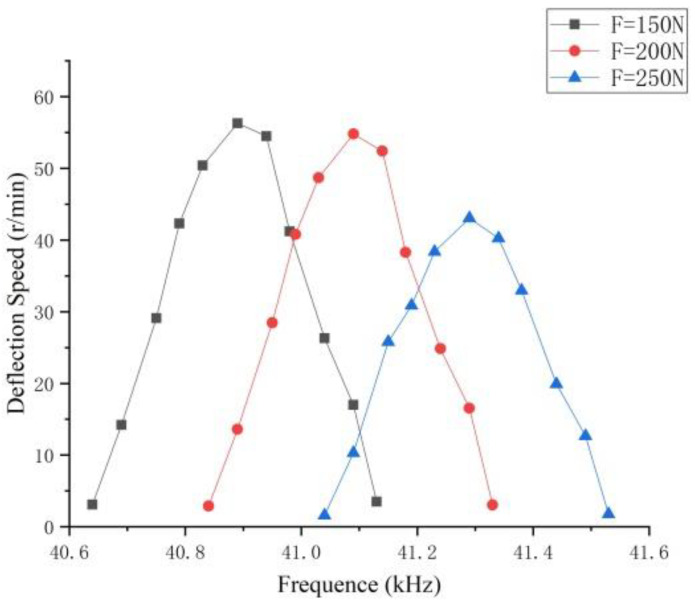
The influence of different pre-pressures on the motor speed.

It can be seen from Figure 15 that the deflection speed of a motor infinitely close to the resonant frequency can reach the extreme value, and the deflection speed of the point farther from the resonance frequency is lower and close to zero. As the pre-pressure increases, the resonance frequency gradually decreases, and the deflection speed also decreases. When the load is constant, the maximum output power is the maximum speed, and the speed extreme point is the optimal pre-pressure point. Therefore, the pre-pressure is the optimal pre-pressure control range in the range of 150 N–200 N, and the deflection speed can reach 56.3 r/min. Beyond this optimal control range, with the increase of the pre-pressure, the stator frequency will rise rapidly, and the friction loss will also increase, which will result in the acceleration of the deflection speed drop and the poor motor operation. Table 3 compares the frequency values under simulation and experiment, showing that the results are similar, and the overall error is less than 5%. Due to the influence of materials and precision in the actual machining process of the motor, the two results are slightly different.

**Figure 16:**
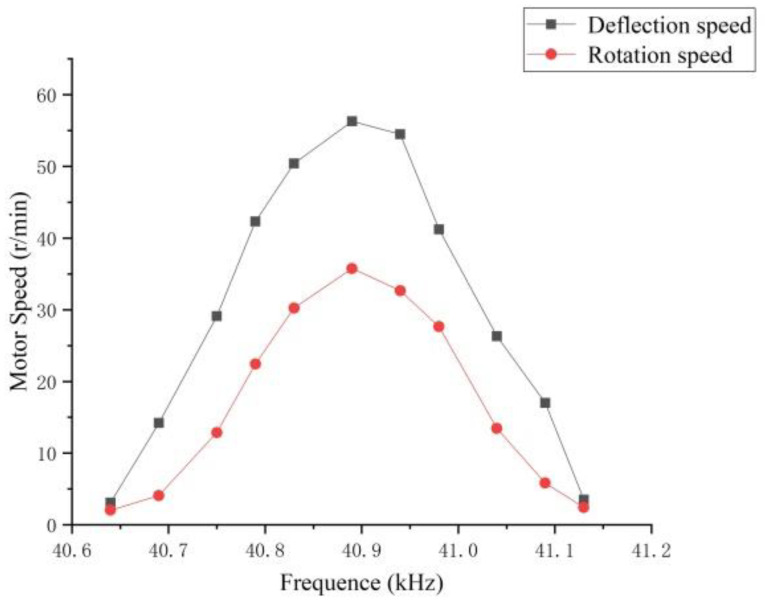
Motor speed curve.

The pre-pressure was set to 150 N, and the deflection and rotation speed of the motor were tested. The result is shown in Figure 16. Both the rotation speed and the deflection speed reach the highest value at the resonance frequency, and the extreme value of the rotation speed is 35.75 r/min; thus, it is proved that the motor can realize multi-degree-of-freedom movement.

**Figure 17:**
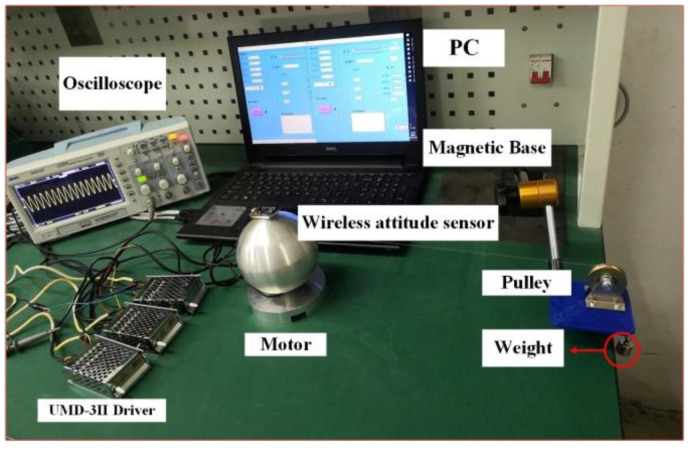
Torque test platform.

**Figure 18:**
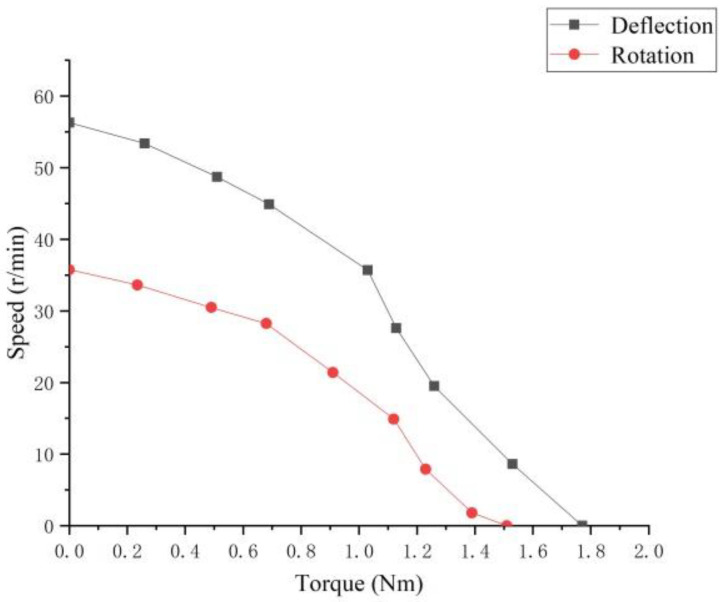
Torque-speed under 150 N preload.

Using the experimental device shown in Figure 17, the rotation torque and the deflection torque of the motor when the preload of the motor is 150 N are measured by using the pulley weighing to change the output torque, and the results are shown in Figure 18. It can be seen that the maximum output torque of the motor is 1.77 Nm, which is in line with the low-speed and high-torque characteristics of the ultrasonic motor.

## 6. Conclusions

This paper studied the pre-compression device of the three-stator ultrasonic motor. The effect of the pre-pressure on the contact model, the position of the constant velocity point, and the modal frequency were theoretically analyzed, and the range of the optimal pre-pressure was determined through experiments, and the motor was tested under the optimal pre-pressure to verify that it could achieve more freedom of movement. The study of preload lays a foundation for the optimization of motor structure and performance, which is conducive to the follow-up study of motor output with larger torque.

## Figures and Tables

**Table 1:** Motor stator structure size parameters.

Outer diameter	30 mm	Tooth circumference angle	3°
Internal diameter	9 mm	Tooth width	5.5 mm
Tooth height	2 mm	Elastic base thickness	2.5 mm
Slotting angle	5°	Piezoelectric ceramic thickness	0.5 mm

**Table 2:** Stator material parameters.

**Material**	Elastic Modulus (E)	Poisson’s Ratio (μ)	Density (ρ)
Phosphor bronze	$1.12\;\times \;10^{11}\;N/m^{2}$	0.33	$8800\;\mathrm{kg}/m^{2}$
PZT-8	$8\;\times \;10^{10}\;N/m^{2}$	0.30	$7600\;\mathrm{kg}/m^{2}$

**Table 3:** Comparison of simulation results and experimental results.

Pre-Pressure	Simulation Frequency	Experiment Frequency	Relative Error
150 N	41,650 Hz	40,895 Hz	1.81%
200 N	42,362 Hz	41,098 Hz	2.98%
250 N	43,408 Hz	41,343 Hz	4.8%
